# C-Terminal Proarginine Vasopressin is Associated with Disease Outcome and Mortality, but not with Delayed Cerebral Ischemia in Critically Ill Patients with an Aneurysmal Subarachnoid Hemorrhage: A Prospective Cohort Study

**DOI:** 10.1007/s12028-022-01540-0

**Published:** 2022-06-25

**Authors:** Jos A. H. van Oers, Dharmanand Ramnarain, Annemarie Oldenbeuving, Piet Vos, Gerwin Roks, Yvette Kluiters, Albertus Beishuizen, Dylan W. de Lange, Harm-Jan de Grooth, Armand R. J. Girbes

**Affiliations:** 1 Department of Intensive Care Medicine, Elisabeth Tweesteden Ziekenhuis, P.O. Box 90151, 5000 LC Tilburg, The Netherlands; 2 Department of Neurology, Elisabeth Tweesteden Ziekenhuis, Tilburg, The Netherlands; 3 Department of Clinical Chemistry, Elisabeth Tweesteden Ziekenhuis, Tilburg, The Netherlands; 4 Department of Intensive Care Medicine, Medisch Spectrum Twente, Enschede, The Netherlands; 5 Department of Intensive Care Medicine, University Medical Centre Utrecht, University Utrecht, Utrecht, The Netherlands; 6 Department of Intensive Care Medicine, Amsterdam University Medical Centre, Vrije Universiteit Medical Centre, Amsterdam, The Netherlands

**Keywords:** CT-proAVP, aSAH, Prognosis

## Abstract

**Background:**

Aneurysmal subarachnoid hemorrhage (aSAH) is an important indication for intensive care unit admission and may lead to significant morbidity and mortality. We assessed the ability of C-terminal proarginine vasopressin (CT-proAVP) to predict disease outcome, mortality, and delayed cerebral ischemia (DCI) in critically ill patients with aSAH compared with the World Federation of Neurological Surgeons (WFNS) score and Acute Physiological and Chronic Health Evaluation IV (APACHE IV) model.

**Methods:**

C-terminal proarginine vasopressin was collected on admission in this single-center, prospective, observational cohort study. The primary aim was to investigate the relationship between CT-proAVP and poor functional outcome at 1 year (Glasgow Outcome Scale score 1–3) in a multivariable logistic regression model adjusted for WFNS and APACHE IV scores. Secondary aims were mortality and DCI. The multivariable logistic regression model for DCI was also adjusted for the modified Fisher scale.

**Results:**

In 100 patients, the median CT-proAVP level was 24.9 pmol/L (interquartile range 11.5–53.8); 45 patients had a poor 1-year functional outcome, 19 patients died within 30 days, 25 patients died within 1 year, and DCI occurred in 28 patients. Receiver operating characteristics curves revealed high accuracy for CT-proAVP to identify patients with poor 1-year functional outcome (area under the curve [AUC] 0.84, 95% confidence interval [CI] 0.77–0.92, *p* < 0.001), 30-day mortality (AUC 0.84, 95% CI 0.76–0.93, *p* < 0.001), and 1-year mortality (AUC 0.79, 95% CI 0.69–0.89, *p* < 0.001). CT-proAVP had a low AUC for identifying patients with DCI (AUC 0.67, 95% CI 0.55–0.79, *p* 0.008). CT-proAVP ≥ 24.9 pmo/L proved to be a significant predictor for poor 1-year functional outcome (odds ratio [OR] 8.04, 95% CI 2.97–21.75, *p* < 0.001), and CT-proAVP ≥ 29.1 pmol/L and ≥ 27.7 pmol/L were significant predictors for 30-day and 1-year mortality (OR 9.31, 95% CI 1.55–56.07, *p* 0.015 and OR 5.15, 95% CI 1.48–17.93, *p* 0.010) in multivariable models with WFNS and APACHE IV scores. CT-proAVP ≥ 29.5 pmol/L was not a significant predictor for DCI in a multivariable model adjusted for the modified Fisher scale (*p* = 0.061).

**Conclusions:**

C-terminal proarginine vasopressin was able to predict poor functional outcome and mortality in critically ill patients with aSAH. Its prognostic ability to predict DCI was low.

**Trial Registration:**

Nederlands Trial Register: NTR4118.

**Supplementary Information:**

The online version contains supplementary material available at 10.1007/s12028-022-01540-0.

## Introduction

Aneurysmal subarachnoid hemorrhage (aSAH), which is caused by a ruptured cerebral aneurysm, is an important indication for intensive care unit (ICU) admission and may lead to significant morbidity and mortality [1–4]. Reported incidences vary from 6 to 9 aSAHs per 100,000 person-years in the general population [1–4]. Approximately 8% of the patients with aSAH die before arrival at the hospital [5]. Case-fatality rates after 1 month are around 25% to 35% [5–7]. Although aSAH occurs at a reasonably young age of 55 years [4], estimates of independence varied between 36 and 55% at assessments up to 12 months after the bleeding [4]. In addition, many patients cannot resume their previous work, have difficulties in relationships, and have an impaired quality of life [8].

The immediate prognosis is determined by the amount of initial intracranial hemorrhage and rebleeding before treatment [1, 3]. To prevent rebleeding, the aneurysm is generally obliterated as soon as possible, either by a neurosurgical procedure, in which a metal clip is placed over the neck of the aneurysm, or by an endovascular procedure, in which platinum coils are inserted inside the aneurysm [1]. Among the secondary complications contributing to morbidity and mortality, delayed cerebral ischemia (DCI) is a major risk factor for bad outcome in patients with aSAH [1, 9–12]. The occurrence of DCI is associated with a 1.5-fold to threefold increase in case-fatality rates after SAH [9, 12]. The World Federation of Neurological Surgeons (WFNS) score was developed to indicate the severity of neurological injury and provide prognostic information regarding outcome in patients with aSAH [13], and the Acute Physiological and Chronic Health Evaluation (APACHE IV) model was developed to assess disease severity or severity of organ dysfunction and predict outcome in critically ill patients [14]. However, finding an accurate prediction of outcome remains difficult and complicates decision making for active treatment aiming at recovery. The modified Fisher scale was designed to predict the risk of DCI in patients with aSAH [13, 15]. It is entirely based on the amount of blood on neuroimaging at initial presentation. Biomarkers, as a surrogate or adjunct of clinical scores, could represent an attractive alternative to predict outcome. C-terminal proarginine vasopressin (CT-proAVP), also termed copeptin, is the C-terminal part of the prohormone of arginine vasopressin (AVP), also termed antidiuretic hormone, which is produced in the hypothalamus and stored in the posterior pituitary [16, 17]. CT-proAVP is stable for days, and therefore measuring CT-proAVP in blood is more feasible for clinical purposes [17]. High levels of CT-proAVP were reported to be predictive of poor outcome in patients with traumatic brain injury [18], intracerebral hemorrhage [19], and ischemic stroke [20]. CT-proAVP levels at admission were highly predictive of poor functional outcome and mortality in three cohort studies with Asian patients with aSAH [21–23] and was a good prognostic marker for DCI [21, 22]. We studied CT-proAVP in Dutch patients with aSAH, as there are differences reported between Asian and White patients regarding incidence and outcome of aSAH [24, 25].

The primary aim of the present study was to investigate the prognostic value of CT-proAVP on admission to predict poor functional outcome after 1 year in critically ill patients with aSAH compared with WFNS and APACHE IV scores. Secondary aims were 30-day and 1-year mortality and DCI.

## Methods

### Study Design and Selection Criteria

In a single-center, prospective, observational cohort study, we enrolled patients with aSAH admitted to the ICU of the Elisabeth Tweesteden hospital (Tilburg, the Netherlands) within 24 h after bleeding from November 2013 until April 2015. The study protocol was approved by the Medisch Ethische Toetsingscommissie Brabant (Tilburg, the Netherlands; trial number NL45096.008.13). Informed consent was obtained from participating patients. Inclusion criteria were adults ≥ 18 years of age, admittance to the ICU with an aSAH within 24 h after bleeding and a CT-proAVP index test on the day of ICU admission. Exclusion criteria for trial participation were recent (< 30 days) ischemic or hemorrhagic stroke, intracerebral hemorrhage without subarachnoid blood, head trauma, acute myocardial infarction, acute exacerbation of chronic obstructive pulmonary disease, sepsis or septic shock, acute pancreatitis, chronic heart failure, and liver cirrhosis. Diagnosis of aSAH was based on clinical symptoms (acute headache, focal neurological deficits, loss of consciousness), presence of blood on computerized tomography (CT) cerebrum or presence of xanthochromia in cerebral spinal fluid in combination with an aneurysm, confirmed by CT angiography or digital subtraction angiography (DSA) [1]. Diagnosis of DCI was based on acute clinical deterioration in the patient’s neurologic condition between three and 14 days after aSAH, assessed by a decrease of at least two points on the Glasgow Coma Scale sum score and/or by the development of new focal neurological deficits, and exclusion of other causes for neurological deterioration [9–12]. In cases of suspected DCI, a CT brain perfusion, CT angiography, or DSA was performed. Other causes for neurological deterioration included increasing hydrocephalus, rebleeding of an aneurysm, epileptic seizure, severe infectious disease with associated decrease in level of consciousness, hypoglycemia (defined as serum glucose < 3 mmol/L), hyponatremia (defined as serum sodium < 125 mmol/L), and metabolic encephalopathy due to renal failure, as indicated by rapidly rising serum urea. We followed the Strengthening the Reporting of Observational Studies in Epidemiology Statement guidelines for reporting observational studies [26]. Included patients and excluded patients are described in the patient flow diagram. A control group consisted of 30 healthy volunteers, all hospital staff, with no vascular risk factors. The primary aim was the prediction of poor functional outcome after 1 year, and secondary aims were the prediction of 30-day and 1-year mortality and prediction of DCI by baseline CT-proAVP. Patients were contacted after 1 year by the research nurse (PV) for a questionnaire by telephone. This questionnaire included the Glasgow Outcome Scale (GOS) [27]. The GOS rated from death (one point) to symptom-free full recovery (five points). GOS scores were dichotomized in good and poor functional outcomes (GOS 4–5 vs. GOS 1–3, respectively). The research nurse was blinded for CT-proAVP levels.

### Procedures

Venous blood was drawn to measure CT-proAVP in the control group at the start of the study. Clinical data and laboratory results were collected on the first day of ICU admission in patients enrolled in the study. The initial CT-cerebrum was classified according to the modified Fisher scale [13, 15]. Blood samples were collected into clot-tubes at admittance. Serum was separated by centrifugation and stored in aliquots at − 80 °C, and Serum CT-proAVP levels were measured afterward using an automated immunofluorescent sandwich assay on a B.R.A.H.M.S. Kryptor Compact Plus analyzer (Thermo Fisher Scientific, Henningsdorf, Germany). The Kryptor measures the signal that is emitted from an immunocomplex by time-resolved amplified cryptate emission. CT-proAVP assays have a lower limit of detection of 0.69 pmol/L. The functional sensitivity (lowest value with an interassay coefficient of variation < 20% as described by the manufacturer) of 1.08 pmol/L. Imprecision of the assay was verified according to the Clinical & Laboratory Standards Institute Evaluation Protocol 17-A, using a low and high sample, measured for 5 days in triplicate. Intracoefficients of variation and intercoefficient of variation values were all ≤ 10% for CT-proAVP.

### Statistical Analysis

To study our hypothesis that CT-proAVP was useful as predictor for poor functional outcome at 1 year, a sample size of 93 patients will have 80% power to calculate sensitivity and specificity for CT-proAVP in patients with aSAH, using a *χ*^2^ test with a 0.05 two-sided significance level. This power calculation was based on CT-proAVP levels in patients with aSAH in a prior study [21]. Normally distributed data were expressed as means (standard deviations); all nonnormally distributed data (Kolmogorov–Smirnov test *p* < 0.05) were expressed as medians (with interquartile ranges) or as number of patients (percentage), when appropriate. Patient characteristics and outcomes were compared using Mann–Whitney *U*-test for skewed distributed continuous variables and *χ*^2^ test for categorical variables. The association between CT-proAVP or severity scores (WFNS and APACHE IV) and poor functional outcome at 1 year, mortality after 30 days and 1 year, and DCI was assessed by using area under the receiver operating characteristics (ROC) curves. Youden’s index analysis was applied to calculate optimal cutoff points. Youden’s index was represented by the following formula: *J* = sensitivity + specificity − 1. Sensitivity, specificity, positive and negative predictive values, and positive and negative likelihood ratios (LR+, LR−) were calculated for CT-proAVP and severity scores. CT-proAVP, WFNS, and APACHE IV were transformed to dichotomous variables (below or equal to and above the cutoff point) and included in univariable logistic regression models to study the effects on outcome, mortality and DCI. Sex, confirmed predictors of outcome and mortality (age, rebleeding), and DCI (modified Fisher scale) were also tested in univariable regression analysis. Variables that yielded *p* values < 0.10 were subsequently included in the multivariable regression analysis. Considering the low number of outcome measures in our study and to avoid overfitting of the model, CT-proAVP was tested with only a limited number of other variables. The model was checked for intercorrelations among the predictor variables by collinearity statistics. CT-proAVP levels were also log transformed to calculate the risk of poor functional outcome at 1 year in a logistic regression analysis formula. All tests were two-sided, and a *p*-value < 0.05 was considered statistically significant. All data were analyzed using a statistical software package (version 24; SPSS Inc., Chicago, IL).

## Results

### Descriptive Characteristics of the Patients

**Fig. 1:**
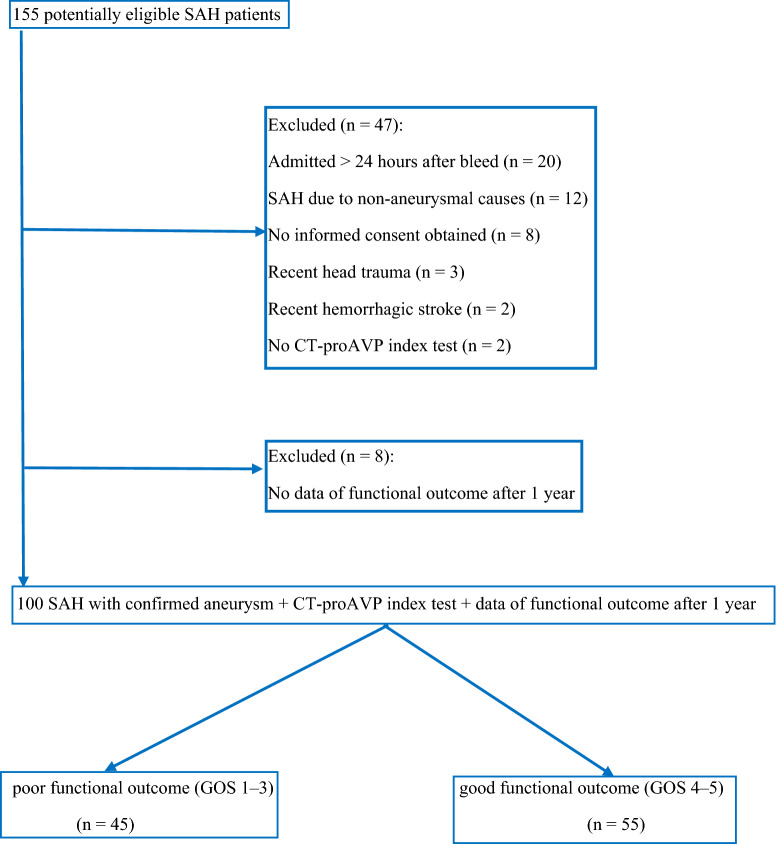
Patient flow diagram. *CT-proAVP* C-terminal proarginine vasopressin, *GOS* Glasgow outcome scale, *SAH* Subarachnoid hemorrhage

During the recruitment period, 155 potentially eligible patients were admitted to the ICU with a presumed diagnosis of aSAH. CT-proAVP levels were measured the first day of admission and data of functional status after 1 year were obtained in 100 patients with SAH with a confirmed aneurysm. The patient flow diagram shows the flow of patients along with the primary end point of 1-year functional outcome (Fig. 1). Table 1 summarizes the clinical and laboratory data of these patients.

**Table 1:** Characteristics of 100 patients with aneurysmal SAH patients

Characteristic	Patients (*N* = 100)
Sex (male/female), (*N*)	24/76
Age, mean (SDsd) (yr)	59.6 (11.8)
GCS at admission, median (IQR)	13 (4–15)
WFNS score at admission, median (IQR)	2 (1–5)
Modified Fisher scale at admission, median (IQR)	3 (1–4)
APACHE IV score ad admission, median (IQR)	49 (31–84)
Aneurysmal location, *N* (%)	
Middle cerebral artery	20 (20%)
Anterior communication artery	40 (40%)
Posterior communication artery	15 (15%)
Posterior inferior cerebelli artery	7 (7%))
Internal carotid artery	3 (3%)
Basilar artery	8 (8%)
Vertebral artery	3 (3%)
Others	4 (4%)
Management, *N* (%)	
Endovascular coiling	74 (74%)
Neurosurgical clipping	15 (15%)
External ventricular/lumbar drainage	35 (35%)
Adverse events during ICU stay, *N* (%)	
Rebleeding	16 (16%)
Acute hydrocephalus	39 (39%)
Clinical deterioration caused by DCI	28 (28%)
Intracerebral hemorrhage	13 (13%)
Intraventricular hemorrhage	22 (22%)
Seizures	10 (10%)
Outcome	
ICU LOS (days), median (IQR) (d)	6 (5–12)
Hospital LOS, (days), median (IQR) (d)	13 (10–21)
30-day mortality, *N* (%) (*N*, %)	19 (19%)
1-year mortality, *N* (%) (*N*, %)	25 (25%)
1-year poor functional outcome (GOS 1–3), *N* (%) (*N*, %)	45 (45%)
Biomarker, median (IQR**)**	
Time from onset bleeding to serum—sampling (in hr)ours, median (IQR**)**	12 (6–17)
Serum CT-proAVP (pmol/L), median (IQR)	24.9 (11.5–53.8)

APACHE IV, Acute Physiology and Chronic Health Evaluation IV; CT-proAVP, C-terminal proarginine vasopressin; DCI, Delayed cerebral ischemia; GCS, Glasgow Coma Scale; GOS, Glasgow outcome scale; ICU, Intensive care unit; IQR, Interquartile range; LOS, Length of stay; SAH, Subarachnoid hemorrhage; SD, Standard deviation; WFNS, World Federation of Neurological Surgeons

### Serum CT-proAVP Level on Admission in Patients with aSAH

Serum CT-proAVP levels at the first day of admission in 100 patients were statistically higher compared with 30 healthy controls, 24.9 pmol/L (11.5–53.8) versus 3.8 pmol/L (3.1–5.3), *p* < 0.001 (Supplemental Fig. 1).

### Association Between CT-proAVP and Poor Functional Outcome at 1 Year

After 1 year, 45 patients had poor functional outcome and 55 had good functional outcome. Patients with poor functional outcome at 1 year had significantly higher CT-proAVP levels compared with patients with good functional outcome at 1 year (53.1 pmol/L [27.4–123.7] vs. 14.3 pmol/L [7.3–26.8], *p* < 0.001). ROC curves revealed high accuracy for CT-proAVP to identify patients with poor functional outcome at 1 year, area under the curve (AUC) 0.84 and 95% confidence interval (CI) 0.77–0.92, *p* < 0.001 (Table 2; Supplemental Fig. 2). When CT-proAVP was combined with WFNS or APACHE IV, the combination of APACHE IV and CT-proAVP yielded the highest AUC (Table 2). CT-proAVP and APACHE IV yielded the highest LR+ (Table 2). Eighty-two percent of the patients with both APACHE IV and CT-proAVP ≥ cutoff point had poor outcome at 1 year, and 90% of the patients with both APACHE IV and CT-proAVP < cutoff point had good outcome after 1 year (Supplemental Table 1). Univariable logistic regression analysis demonstrated that CT-proAVP ≥ 24.9 pmol/L and APACHE IV ≥ 44 points had the strongest association with increased risk of poor functional outcome at 1 year compared with patients with values below the cutoff point (Table 3). CT-proAVP, WFNS, APACHE IV and age were included in a multivariable logistic regression model. CT-proAVP ≥ 24.9 pmol/L proved to be a significant predictor for poor functional outcome at 1 year (odds ratio [OR] 8.04, 95% CI 2.97–21.75, *p* < 0.001) (Table 3). There was moderate correlation among predictor variables, variance inflation factors (VIF) of APACHE IV and WNFS were 4.74 and 4.04, respectively (Supplemental Table 2). The model was tested for interaction between APACHE IV and WFNS scores in a post-hoc analysis. An APACHE IV × WFNS interaction term made no significant contribution to the multivariable model (*p* = 0.097). The risk of poor functional outcome at 1 year could be calculated by the following logistic regression analysis formula: ln (*p*/1 − *p*) =  − 7.618 + 1.386*α*_1_ + 0.047*α*_2_, in which *α*_1_ is ln (CT-proAVP level) and *α*_2_ is APACHE IV score, *p* is the probability of poor functional outcome at 1 year, and *p*/1 − *p* is the odds of developing poor functional outcome after 1 year. WFNS score and age were not significant variables in the multivariable regression model.

**Table 2:** Prediction of poor outcome at 1-year poor outcome by clinical score and CT-proAVP

Parameter	AUC (95% CI)	*p* value	Cutoff	Sens (%)	Spec (%)	PPV (%)	NPV (%)	LR+	LR−
APACHE IV	0.79 (0.69–0.88)	< 0.001	44	80	64	64	80	2.22	0.31
WFNS	0.69 (0.57–0.80)	0.001	3	62	67	61	69	1.88	0.57
CT-proAVP	0.84 (0.77–0.92)	< 0.001	24.9	78	73	70	80	2.89	0.30
APACHE IV + CT-proAVP	0.87 (0.80–0.94)	< 0.001	NA	NA	NA	NA	NA	NA	NA
WFNS + CT-proAVP	0.84 (0.76–0.92)	< 0.001	NA	NA	NA	NA	NA	NA	NA

APACHE IV, Acute Physiology and Chronic Health Evaluation IV; AUC, Area under the curve; CI, Confidence interval; CT-proAVP, C-terminal proarginine vasopressin; LR−, Negative likelihood ratio; LR+, Positive likelihood ratio; NA, Not applicable; NPV, Negative predictive value; PPV, Positive predictive value; Sens, Sensitivity; Spec, Specificity; WFNS, World Federation of Neurological Surgeons

**Table 3:** Univariable and multivariable logistic regression analysis of factors predicting 1-year poor functional outcome at 1 year

Parameter	Univariable analysis		Multivariable analysis	
	OR (95% CI)	*p* value	OR (95% CI)	*p* value
Age	1.04 (0.99–1.07)	0.060	1.02 (0.98–1.07)	0.359
Sex (female vs. male)	1.20 (0.47–3.02)	0.707	–	–
Rebleeding	1.71 (0.58–5.04)	0.327	–	–
APACHE IV at admission				
< 44	1.0 (Reference)		1.0 (Reference)	
≥ 44	7.00 (2.81–17.46)	< 0.001	4.26 (1.04–17.54)	0.045
WFNS at admission				
< 3	1.0 (Reference)		1.0 (Reference)	
≥ 3	3.39 (1.48–7.73)	0.004	1.34 (0.35–5.16)	0.675
Serum CT-proAVP (pmol/L) at admission				
< 24.9	1.0 (Reference)		1.0 (Reference)	
≥ 24.9	9.33 (3.72–23.42)	< 0.001	8.04 (2.97–21.75)	< 0.001

APACHE IV, Acute Physiology and Chronic Health Evaluation IV; CI, Confidence interval; CT-proAVP, C-terminal proarginine vasopressin; OR, Odds ratio, WFNS; World Federation of Neurological Surgeons

### Association Between CT-proAVP and 30-Day and 1-Year Mortality

Nineteen patients died of aSAH in 30 days and 25 patients died within 1 year. Nonsurvivors at 30 days and 1 year had significant higher concentrations of CT-proAVP the first day of admission than survivors (87.8 [30.1–228.8] vs. 18.4 pmol/L [9.7–39.9], *p* < 0.001 for 30-day mortality and 58.4 [29.0–163.8] vs. 18.4 [8.8–38.7], *p* < 0.001 for 1-year mortality). ROC curves revealed high accuracy for CT-proAVP to identify both patients with 30-day mortality (AUC 0.84, 95% CI 0.76–0.93, *p* < 0.001) and 1-year mortality (AUC 0.79, 95% CI 0.69–0.89, *p* < 0.001) (Table 4; (Supplemental Figs. 3, 4). The predictive value of CT-proAVP was lower than those of WFNS (30-day mortality) and APACHE IV (30-day and 1-year mortality). When CT-proAVP was combined with WFNS or APACHE IV, the combination of APACHE IV and CT-proAVP yielded the highest AUC (Table 4). APACHE IV yielded the highest LR+ for the prediction of 30-day and 1-year mortality (Table 4). All (100%) patients with both APACHE IV and CT-proAVP < cutoff point survived 30 days and 1 year (Supplemental Tables 3, 4). A smaller part (65% and 61%, respectively) of the patients with both APACHE IV and CT-proAVP ≥ cutoff point died in 30 days and 1 year (Supplemental Tables 3, 4). Univariable logistic regression analysis demonstrated that APACHE IV above the cutoff points had the strongest association with increased risk of 30-day and 1-year mortality compared with patients with values below the cutoff point (Table 5). CT-proAVP, WFNS, APACHE IV, age, and rebleeding were included in a multivariable logistic regression model for 30-day mortality and all, but rebleeding were included in further multivariable analysis for 1-year mortality. CT-proAVP ≥ 29.1 pmol/L and 27.7 pmol/L proved to be a significant predictor for 30-day and 1-year mortality (OR 9.31, 95% CI 1.55–56.07, *p* 0.015 and OR 5.15, 95% CI 1.48–17.93, *p* = 0.010), but not as strong predictor as APACHE IV in predicting 30-day and 1-year mortality (OR 18.27, 95% CI 1.19–281.53, *p* = 0.037 and OR 10.25, 95% CI 1.45–72.48, *p* = 0.020) (Table 5). There was a moderate correlation between predictor variables, APACHE IV and WFNS had the highest VIFs (Supplemental Tables 5, 6). The models were tested for interaction between APACHE IV and WFNS scores in a post-hoc analysis. An APACHE IV × WFNS interaction term made no significant contribution to the multivariable model of 30-day and 1-year mortality (*p* = 0.276 and *p* = 0.233, respectively).

**Table 4:** Prediction of 30-day and 1-year mortality by clinical score or CT-proAVP

Mortality	AUC (95% CI)	*p* value	Cutoff	Sens (%)	Spec (%)	PPV (%)	NPV (%)	LR+	LR−
30-day mortality									
APACHE IV	0.94 (0.89–0.99)	< 0.001	70	95%	80%	53%	99%	4.75	0.06
WFNS	0.88 (0.81–0.95)	< 0.001	3	95%	65%	39%	98%	2.71	0.08
CT-proAVP	0.84 (0.76–0.93)	< 0.001	29.1	84%	64%	36%	95%	2.33	0.25
APACHE IV + CT-proAVP	0.94 (0.90–0.98)	< 0.001	NA	NA	NA	NA	NA	NA	NA
WFNS + CT-proAVP	0.92 (0.87–0.98)	< 0.001	NA	NA	NA	NA	NA	NA	NA
1-year mortality									
APACHE IV	0.87 (0.80–0.95)	< 0.001	54	88%	71%	50%	95%	3.03	0.17
WFNS	0.77 (0.66–0.89)	< 0.001	3	80%	65%	44%	91%	2.29	0.33
CT-proAVP	0.79 (0.69–0.89)	< 0.001	27.7	80%	64%	43%	91%	2.22	0.31
APACHE IV + CT-proAVP	0.88 (0.81–0.95)	< 0.001	NA	NA	NA	NA	NA	NA	NA
WFNS + CT-proAVP	0.82 (0.73–0.92)	< 0.001	NA	NA	NA	NA	NA	NA	NA

APACHE IV, Acute Physiology and Chronic Health Evaluation IV; AUC, Area under the curve; CI, Confidence interval; CT-proAVP, C-terminal proarginine vasopressin; LR−, Negative likelihood ratio; LR+, Positive likelihood ratio; NA, Not applicable; NPV, Negative predictive value; PPV, Positive predictive value; Sens, Sensitivity; Spec, Specificity; WFNS, World Federation of Neurological Surgeons

**Table 5:** Univariable and multivariable logistic regression analysis of factor predicting 30-day and 1-year mortality

Mortality	Univariable analysis		Multivariable analysis	
	OR (95% CI)	*p* value	OR (95% CI)	*p* value
30-day mortality				
Age	1.04 (0.99–1.09)	0.094	1.04 (0.97–1.12)	0.246
Sex (female vs. male)	1.87 (0.49–7.05)	0.357	–	–
Rebleeding	3.28 (1.02–10.58)	0.047	1.88 (0.32–11.06)	0.488
APACHE IV at admission				
< 70	1.0 (Reference)		1.0 (Reference)	
≥ 70	73.13 (9.08–589.23)	< 0.001	18.27 (1.19–281.53)	0.037
WFNS score at admission				
< 3	1.0 (Reference)		1.0 (Reference)	
≥ 3	34.07 (4.32–268.68)	0.001	4.85 (0.22–106.80)	0.317
Serum CT-proAVP at admission (pmol/L)				
< 29.1	1.0 (Reference)		1.0 (Reference)	
≥ 29.1	9.56 (2.57–35.59)	0.001	9.31 (1.55–56.07)	0.015
1-year mortality				
Age	1.05 (1.01–1.10)	0.026	1.05 (0.99–1.11)	0.062
Sex (female vs. male)	1.36 (0.45–4.12)	0.590	–	–
Rebleeding	2.05 (0.66–6.38)	0.214	–	–
APACHE IV at admission				
< 54	1.0 (Reference)		1.0 (Reference)	
≥ 54	17.67 (4.79–65.13)	< 0.001	10.25 (1.45–72.48)	0.020
WFNS score at admission				
< 3	1.0 (Reference)		1.0 (Reference)	
≥ 3	7.54 (2.54–22.41)	0.004	1.54 (0.24– 9.88)	0.649
Serum CT-proAVP (pmol/L) at admission				
< 27.7	1.0 (Reference)		1.0 (Reference)	
≥ 27.7	7.11 (2.40–21.10)	< 0.001	5.15 (1.48–17.93)	0.010

APACHE IV, Acute Physiology and Chronic Health Evaluation IV; CI, Confidence interval; CT-proAVP, C-terminal proarginine vasopressin; OR, Odds ratio; WFNS, World Federation of Neurological Surgeons

### Association Between CT-proAVP and DCI

Twenty-eight patients with aSAH experienced clinical signs of DCI during their ICU stay. Patients with DCI had significantly higher concentrations of CT-proAVP concentrations the first day of admission than patients without DCI (51 pmol/L [15.9–116.1] vs. 20.8 pmol/L [9.4–40.0], *p* 0.008). However, CT-proAVP had a low accuracy rate for identifying patients with DCI in the ROC analysis (AUC 0.67, 95% CI 0.55–0.79, *p* = 0.008) (Table 6; Supplemental Fig. 5). WFNS, APACHE IV, and modified Fisher scale had comparably low accuracy rates for predicting DCI, with low AUCs in ROC analysis (Table 6; Supplemental Fig. 5). An optimal cutoff point was calculated for CT-proAVP and modified Fisher scale. No optimal cutoff points could be calculated for WFNS and APACHE IV scores, and they were not tested in the multivariable model. CT-proAVP yielded a low LR+ for the prediction of DCI. Both CT-proAVP and modified Fisher scale were tested in a multivariable logistic regression model. CT-proAVP ≥ 29.5 pmol/L was not a significant predictor for DCI in a multivariable model adjusted for the modified Fisher scale (OR 2.51, 95% CI 0.96–6.56, *p* = 0.061) (Table 7). There was no correlation between CT-proAVP and modified Fisher scale (VIF 1.09) (Supplemental Table 7).

**Table 6:** Prediction of delayed cerebral ischemia during hospitalization by clinical score or CT-proAVP

Parameter	AUC (95% CI)	*p* value	Cutoff	Sens (%)	Spec (%)	PPV (%)	NPV (%)	LR+	LR−
APACHE IV	0.60 (0.48–0.71)	0.136	NA	NA	NA	NA	NA	NA	NA
WFNS	0.51 (0.38–0.64)	0.902	NA	NA	NA	NA	NA	NA	NA
Modified Fisher scale	0.65 (0.54–0.77)	0.018	3.0	82	54	41	89	3.72	0.66
CT-proAVP	0.67 (0.55–0.79)	0.008	29.5	64	65	42	83	1.83	0.55
APACHE IV + CT-proAVP	0.61 (0.48–0735)	0.105	NA	NA	NA	NA	NA	NA	NA
WFNS + CT-proAVP	0.62 (0.49–0.74)	0.071	NA	NA	NA	NA	NA	NA	NA
Modified Fisher scale + CT-proAVPproAVP	0.70 (0.59–0.81)	0.02	NA	NA	NA	NA	NA	NA	NA

APACHE IV, Acute Physiology and Chronic Health Evaluation IV; AUC, Area under the curve; CI, Confidence interval; CT-proAVP, C-terminal proarginine vasopressin; LR−, Negative likelihood ratio; LR+, Positive likelihood ratio; NPV, Negative predictive value; PPV, Positive predictive value; Sens, Sensitivity; Spec, Specificity; WFNS, World Federation of Neurological Surgeons

**Table 7:** Univariable and multivariable logistic regression analysis of factors predicting DCI

Parameter	Univariable analysis		Multivariable analysis	
	OR (95% CI)	*p* value	OR (95% CI)	*p* value
Age	1.01 (0.97–1.04)	0.786	–	–
Sex (female vs. male)	1.65 (0.55–4.95)	0.373	–	–
Modified Fisher scale at admission				
< 3	1.0 (Reference)		1.0 (Reference)	
≥ 3	5.44 (1.86–15.89)	0.002	4.41 (1.47–13.24)	0.008
Serum CT-proAVP (pmol/L) at admission				
< 29.5	1.0 (Reference)		1.0 (Reference)	
≥ 29.5	3.38 (1.36–8.43)	0.009	2.51 (0.96–6.56)	0.061

CI, Confidence interval; CT-proAVP, C-terminal proarginine vasopressin; OR, Odds ratio

## Discussion

We reported two main findings. First, CT-proAVP had a high level of accuracy in identifying critically ill patients with aSAH with poor functional outcome and was a significant predictor in a multivariable logistic regression model including WFNS and APACHE IV scores. Combining CT-proAVP with APACHE IV significantly improved the prognostic accuracy for predicting poor functional outcome at 1 year. Eighty-two percent of the patients with both APACHE IV and CT-proAVP ≥ cutoff point had a poor outcome after 1 year. Secondly, CT-proAVP levels also had a high level of accuracy in identifying critically ill patients with aSAH who died in 30 days and 1 year, but CT-proAVP levels were not predictive of DCI during ICU stay. APACHE IV performed better than the WFNS score in predicting outcome and mortality in our study. The WFNS score was based on the Glasgow Coma Scale and the presence of focal neurological deficit [13], but it can be difficult to assess the neurological status due to sedation or impaired consciousness. A possible explanation for the good performance of APACHE IV in predicting outcome and mortality was because it captured the physiologic stress of aSAH by the physiological subscore of APACHE IV, assessing the degree of acute illness. Age and comorbidities are other known predictors of outcome and mortality and are covered by the Age and Chronic Health section of the APACHE IV score. However, incorporation of APACHE IV model in daily routine was hampered due to its complexity. Finding an easily obtainable biomarker, as alternative or adjunct of clinical scores, able to identify patients with worst outcome may help early risk assessment and may provide further insights into pathophysiological mechanisms. It might be argued that especially patients with highest values CT-proAVP would benefit from extended ICU therapy. On the other hand, patients with lower CT-proAVP values have a higher chance of good functional outcome at 1 year and could be discharged from the ICU to the general ward at an earlier stage.

Our findings of good ability of baseline CT-proAVP levels in serum to predict poor functional outcome and mortality are in line with other studies [21–23]. CT-proAVP, measured during the first day of admission, was frequently studied in Asian patients with aSAH [21–23]. Baseline CT-proAVP levels and WFNS scores in these studies were quite comparable with our study population [21, 22]. CT-proAVP levels at baseline were also strongly correlated with WFNS scores, suggesting CT-proAVP as a robust indicator of neurological outcome following aSAH [22, 28]. In contrast to our findings, combining CT-proAVP with WFNS scores further improved the predictive performance of WFNS scores for poor outcome and mortality in several studies [21, 22]. Elevated baseline CT-proAVP levels correlated with clinical deterioration caused by DCI in several studies [21, 22] and CT-proAVP was an independent predictor of clinical deterioration caused by DCI in logistic regression models [21]. This was considered an important finding, as DCI is the most important treatable determinant of poor outcome after aSAH [29]. Unexpectedly, CT-proAVP levels demonstrated a low ability to predict DCI in our study. We used the term DCI to address clinical deterioration caused by DCI [11]. There are some disadvantages of this clinical diagnosis. The clinical spectrum of DCI is wide. Typical features are neurological deficits or decrease in levels of consciousness. However, neck stiffness, fever, or mutism have also been reported as clinical signs of DCI in some studies [11]. A proportion of patients with aSAH are comatose or sedated. Last, clinical deterioration is a diagnosis per exclusionism. Zhu et al. [21] and Zheng et al. [22] used the term “cerebral vasospasm” for describing clinical deterioration from DCI, but the term “vasospasm” should be reserved for the results of radiological tests (either CT angiography, DSA, or Magnetic Resonance Angiography) [11]. We studied CT-proAVP levels measured once at baseline and the occurrence of DCI during ICU stay. However, significant differences in plasma CT-proAVP levels between patients with DCI and patients without DCI and at different time points were only found from day seven, when consecutive CT-proAVP levels were collected for DCI prediction the first 2 weeks in patients with aSAH [30], suggesting a dynamic secretion of CT-proAVP which necessitates serial CT-proAVP measurements to more accurately predict DCI [30]. In addition, it was found that increased CT-proAVP levels in cerebrospinal fluid were also associated with DCI in patients with aSAH [31].

CT-proAVP, the C-terminal part of the prohormone of AVP, is produced in the hypothalamus [16, 17]. AVP contributes to the regulation of osmotic and cardiovascular homeostasis [16, 17]. AVP is stimulated by different stressors. AVP potentiates the action of the corticotrophin-releasing hormone and leads downstream to release adrenocorticotrophic hormones and produce cortisol [16], reflecting the individual stress response at the hypothalamic level [16]. CT-proAVP concentrations mirror the concentrations of AVP [17]. CT-proAVP is stable for days, and therefore measuring CT-proAVP in blood is more feasible for clinical purposes [17]. CT-proAVP is known to have prognostic value in various diseases, as it reflects disease severity and the chance of recovery [18–20]. Therefore, it has been hypothesized that the close relationship of CT-proAVP levels to the degree of activation of the stress axis is the basis of its usefulness as prognostic biomarker in patients with aSAH [16]. Baseline CT-proAVP levels were predictive of outcome and mortality in our study but not for DCI. The exact underlying pathophysiological mechanisms of DCI are multifactorial and not fully understood [9–12]. Animal studies suggest that AVP could be involved in the development of DCI [32] and ischemic brain edema [33]. Intracisternal injection of AVP induced acute vasospasm in a model of SAH in rats [32]. Treatment with vasopressin receptor antagonists reduced the infarction volume in an embolic focal ischemia model in rats [33].

Some limitations of our study need to be addressed. First, we did a single-center, prospective, observational study in a cohort of patients with aSAH admitted to the ICU within 24 h after bleeding from November 2013 until April 2015. Results of single-center studies are determined by the case-mix (which varies with the age profile and comorbidities of the patients) and resources (number of physicians, nurse-to-patient ratio) of the particular ICU [34]. We must be careful about extrapolating these results to the general population. Second, we collected baseline CT-proAVP levels and did not collect serial CT-proAVP levels during ICU stay. Third, by selecting 100 patients out of 155 potential eligible critically ill patients with SAH on the basis of inclusion criteria, an index test CT-proAVP at admission and data of functional outcome after 1 year, we introduced selection bias. We believe that both observational and selection bias in our study may have led to potential underestimation of the prognostic performance of CT-proAVP and therefore plan to conduct a larger multicenter prospective observational study with serial CT-proAVP measurements in the near future.

CT-proAVP as single baseline value will always oversimplify prognostic assessment, and therefore CT-proAVP is meant, rather than to supersede, to complement clinician’s judgment. Prognosis cannot be based on a biomarker alone, even when it is highly sensitive and specific.

## Conclusions

We conclude that single baseline CT-proAVP was able to predict poor functional outcome at 1 year in critically ill patients with aSAH. Baseline CT-proAVP as adjunct to the APACHE IV model may help clinicians to identify patients at a higher risk of poor outcome. CT-proAVP levels also achieved a high level of accuracy in predicting mortality, but the prognostic ability of single baseline CT-proAVP to predict DCI during ICU stay was low.

## Supplementary Information

Below is the link to the electronic supplementary material.

Supplementary file1 (PDF 607 kb)
