# Supplementary material for: C-Terminal Proarginine Vasopressin is Associated with Disease Outcome and Mortality, but not with Delayed Cerebral Ischemia in Critically Ill Patients with an Aneurysmal Subarachnoid Hemorrhage: A Prospective Cohort Study
Source: Neurocrit Care. 2022 Jun 25;37(3):678–88. doi: 10.1007/s12028-022-01540-0 (PMC9672019; doi:10.1007/s12028-022-01540-0)
Supplement: Supplementary file 1 — Supplementary file1 (PDF 607 kb) [file 12028_2022_1540_MOESM1_ESM.pdf]

Appendix

C-terminal pro-arginine vasopressin is associated with disease outcome and mortality, but not with delayed cerebral ischemia in critically ill patients with an aneurysmal subarachnoid hemorrhage: a prospective cohort study

Jos AH van Oers, Dharmanand Ramnarain, Annemarie Oldenbeuving, Piet Vos, Gerwin Roks, Yvette Kluiters, Albertus Beishuizen, Dylan W de Lange, Harm-Jan de Grooth, Armand RJ Girbes

Supplemental table 1……….…………………………………………………………….…………………page 2

Supplemental table 2……………………………………………………………………………………….page 3

Supplemental table 3………………………………………….……………………………………………page 4

Supplemental table 4………………………………………….……………………………………………page 5

Supplemental table 5………………………………………….……………………………………………page 6

Supplemental table 6………………………………………….……………………………………………page 7

Supplemental table 7………………………………………….……………………………………………page 8

Supplemental figure 1……………………………………………………………………………….……..page 9

Supplemental figure 2………………………………………………………………….……………………page 10

Supplemental figure 3…………………………………………………………………..……..……….page 11

Supplemental figure 4………………………………………………………………..……….……..…page 12

Supplemental figure 5……………………………………………………..……………….….….…page 13

Supplemental Table 1

Predicted probability of one-year poor functional outcome for combinations CT-proAVP and APACHE IV

	APACHE IV < 44	APACHE IV ≥ 44
CT-proAVP < 24.9 pmol/L	10%	36%
CT-proAVP ≥ 24.9 pmol/L	40%	82%

Legends: APACHE IV: Acute physiology and chronic health evaluation IV, CT-proAVP: C-terminal pro-arginine vasopressin. Values indicate the probability (in percentage) of one-year poor functional outcome given the ranges of CT-proAVP and APACHE IV.

Supplemental Table 2

Collinearity statistics of predictor variables in multivariable logistic regression model for one-year poor functional outcome

	**Variance Inflation Factor (VIF)**
Age	1.19
APACHE IV	4.74
WFNS	4.04
CT-proAVP	1.30

Supplemental Table 3

Predicted probability of 30-day mortality for combinations of CT-proAVP and APACHE IV

	APACHE IV < 70	APACHE IV ≥ 70
CT-proAVP < 29.1 pmol/L	0%	21%
CT-proAVP ≥ 29.1 pmol/L	11%	65%

Legends: APACHE IV: Acute physiology and chronic health evaluation IV, CT-proAVP: C-terminal pro-arginine vasopressin. Values indicate the probability (in percentage) of 30-day mortality given the ranges of CT-proAVP and APACHE IV.

Supplemental Table 4

Predicted probability of one-year mortality for combinations of CT-proAVP and APACHE IV (Values indicate the probability (in percentage) of one-year mortality given the ranges of CT-proAVP and APACHE IV)

	APACHE IV < 54	APACHE IV ≥ 54
CT-proAVP < 27.7 pmol/L	0%	27%
CT-proAVP ≥ 27.7 pmol/L	44%	61%

Legends: APACHE IV: Acute physiology and chronic health evaluation IV, CT-proAVP: C-terminal pro-arginine vasopressin. Values indicate the probability (in percentage) of one-year mortality given the ranges of CT-proAVP and APACHE IV.

Supplemental Table 5

Collinearity statistics of predictor variables in multivariable logistic regression model for 30-day mortality

	**Variance Inflation Factor (VIF)**
Age	1.20
Rebleeding	1.09
APACHE IV	4.75
WFNS	4.06
CT-proAVP	1.31

Supplemental Table 6

Collinearity statistics of predictor variables in multivariable logistic regression model for one-year mortality

	**Variance Inflation Factor (VIF)**
Age	1.19
APACHE IV	4.74
WFNS	4.04
CT-proAVP	1.30

Supplemental Table 7

Collinearity statistics of predictor variables in multivariable logistic regression model for DCI

	**Variance Inflation Factor (VIF)**
Modified Fisher scale	1.09
CT-proAVP	1.09

Supplemental figure 1

Serum CT-proAVP concentrations in aneurysmal SAH patients and healthy controls

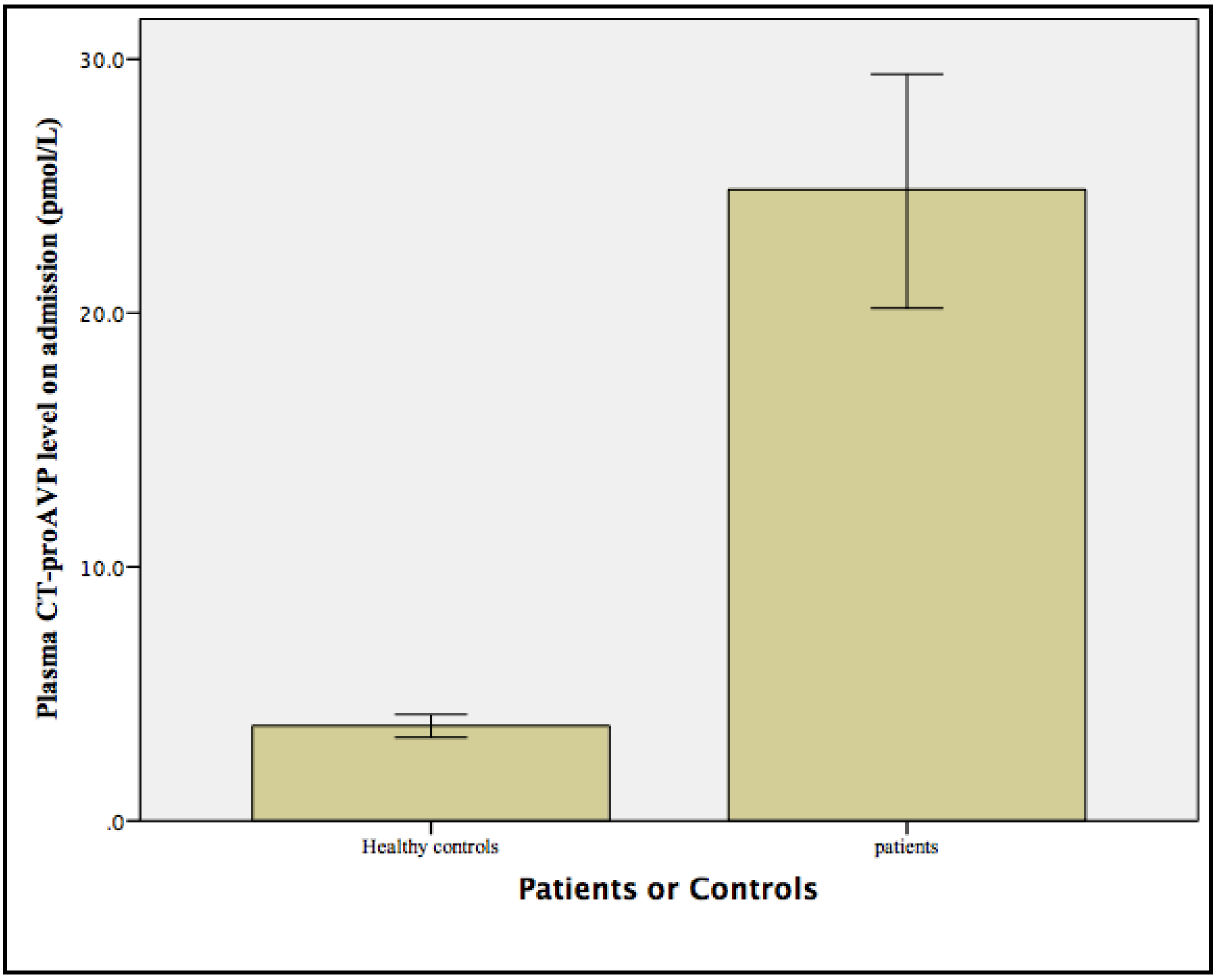

Legends: CT-proAVP: C-terminal pro-arginine vasopressin.

Supplemental figure 2

Receiver operating characteristics curve for clinical score and CT-proAVP in predicting one-year poor functional outcome.

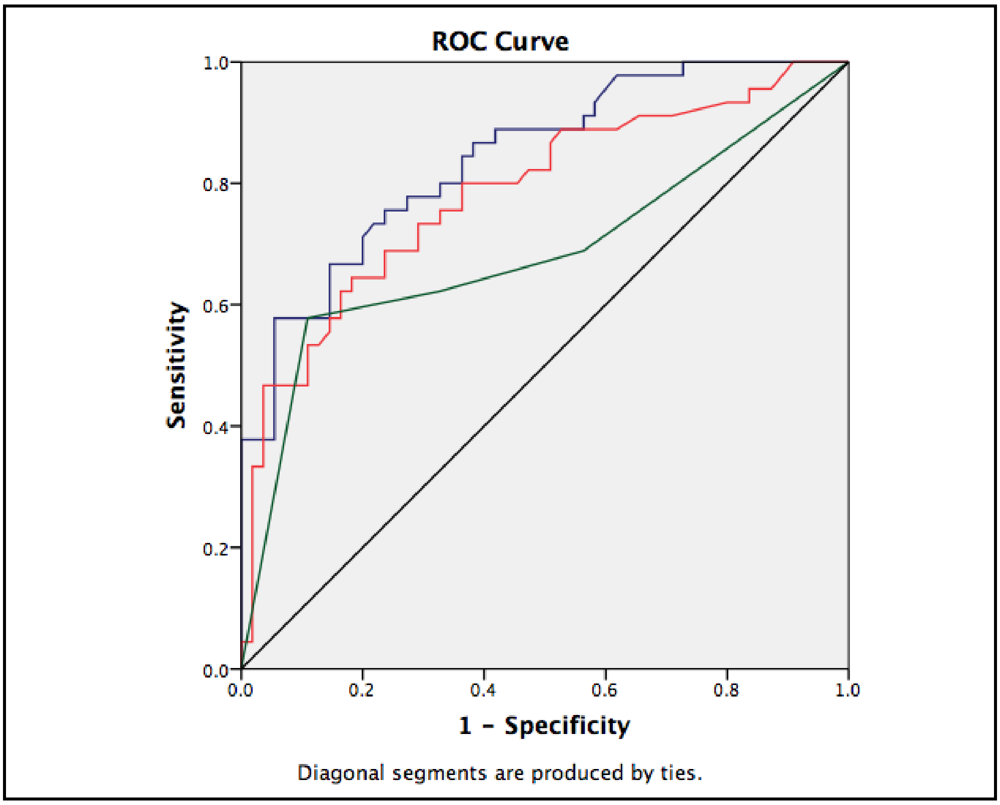

References: Red line = APACHE IV (AUC: 0.79, 95% CI: 0.69 – 0.88, *p* <0.001), green line = WFNS (AUC: 0.69, 95% CI: 0.57 – 0.80, *p* 0.001), blue line = CT-proAVP (AUC: 0.84, 95% CI: 0.77 - 0.92, *p* < 0.001) and black line = reference line.

Legends: APACHE IV: Acute physiology and chronic health evaluation IV, WFNS: World Federation of Neurological Surgeons score, CT-proAVP: C-terminal pro-arginine vasopressin.

Supplemental figure 3

Receiver operating characteristics curve for clinical score and CT-proAVP in predicting 30-day mortality

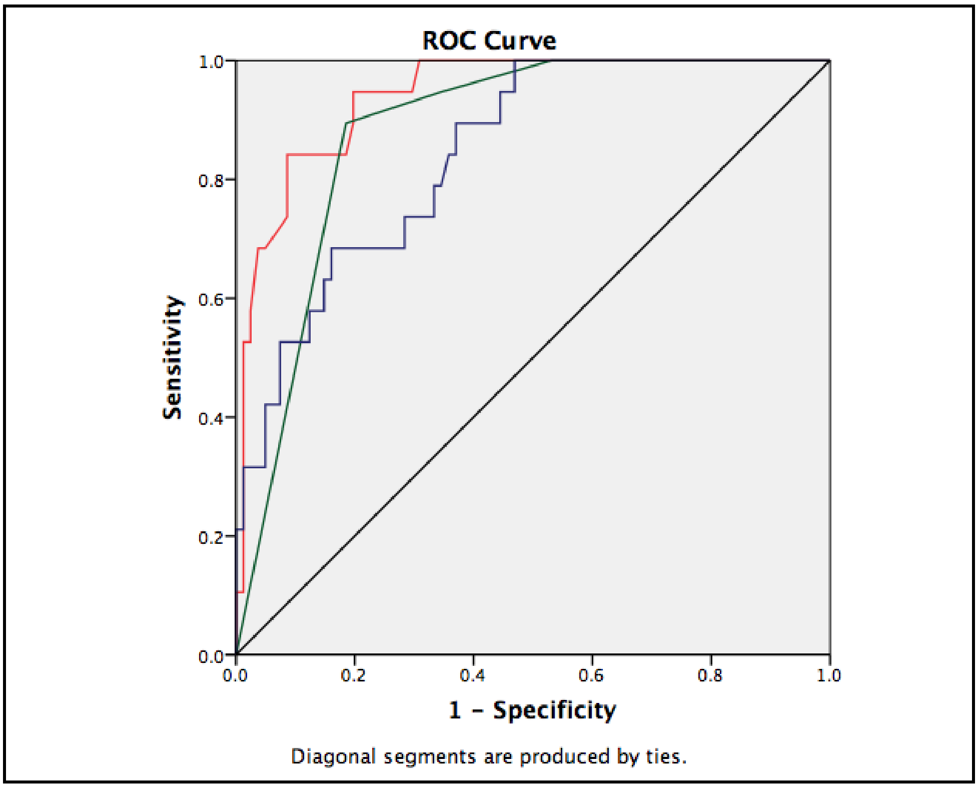

References: Red line = APACHE IV (AUC: 0.94, 95% CI: 0.89 – 0.99, p <0.001), green line = WFNS (AUC: 0.88, 95% CI: 0.81 – 0.95, p < 0.001), blue line = CT-proAVP (AUC: 0.84, 95% CI: 0.76 - 0.93, p < 0.001) and black line = reference line.

Legends: APACHE IV: Acute physiology and chronic health evaluation IV, WFNS: World Federation of Neurological Surgeons score, CT-proAVP: C-terminal pro-arginine vasopressin.

Supplemental figure 4

Receiver operating characteristics curve for clinical score and CT-proAVP in predicting one-year mortality

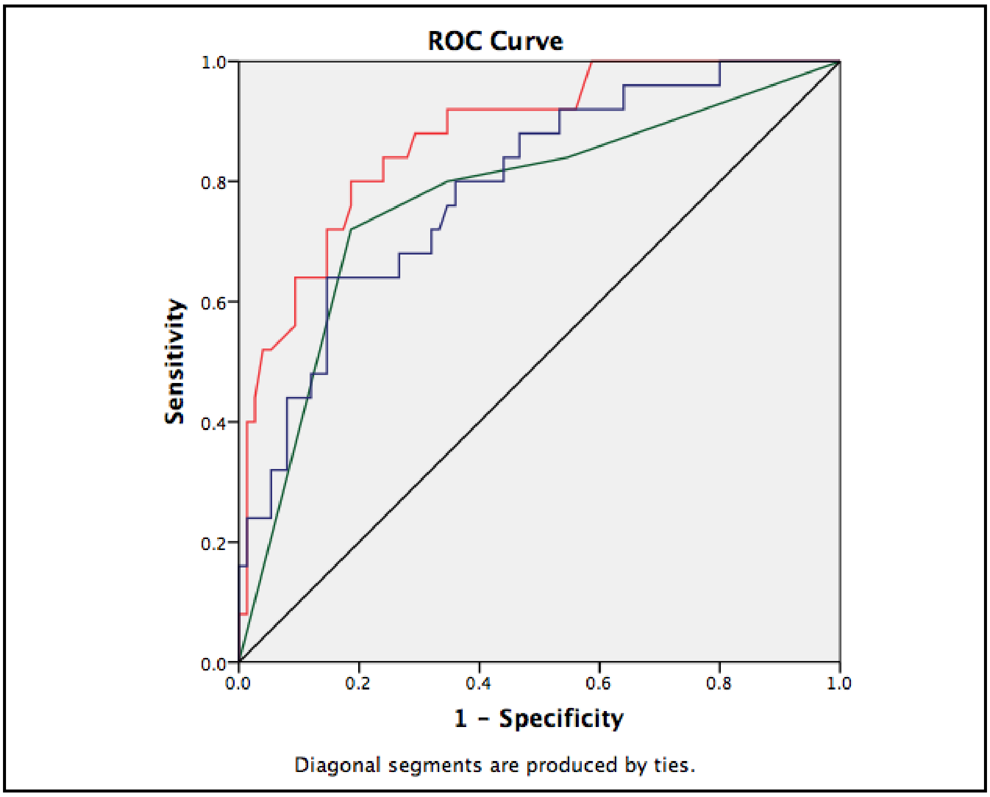

Receiver operating characteristics curve for clinical score and CT-proAVP in predicting 1-year mortality. References: Red line = APACHE IV (AUC: 0.87, 95% CI: 0.80 – 0.98, p <0.001), green line = WFNS (AUC: 0.77, 95% CI: 0.66 – 0.89, p < 0.001), blue line = CT-proAVP (AUC: 0.79, 95% CI: 0.69 - 0.89, p < 0.001) and black line = reference line.

Legends: APACHE IV: Acute physiology and chronic health evaluation IV, WFNS: World Federation of Neurological Surgeons score, CT-proAVP: C-terminal pro-arginine vasopressin.

Supplemental figure 5

Receiver operating characteristics curve for clinical score and CT-proAVP in predicting delayed cerebral ischemia during hospitalization

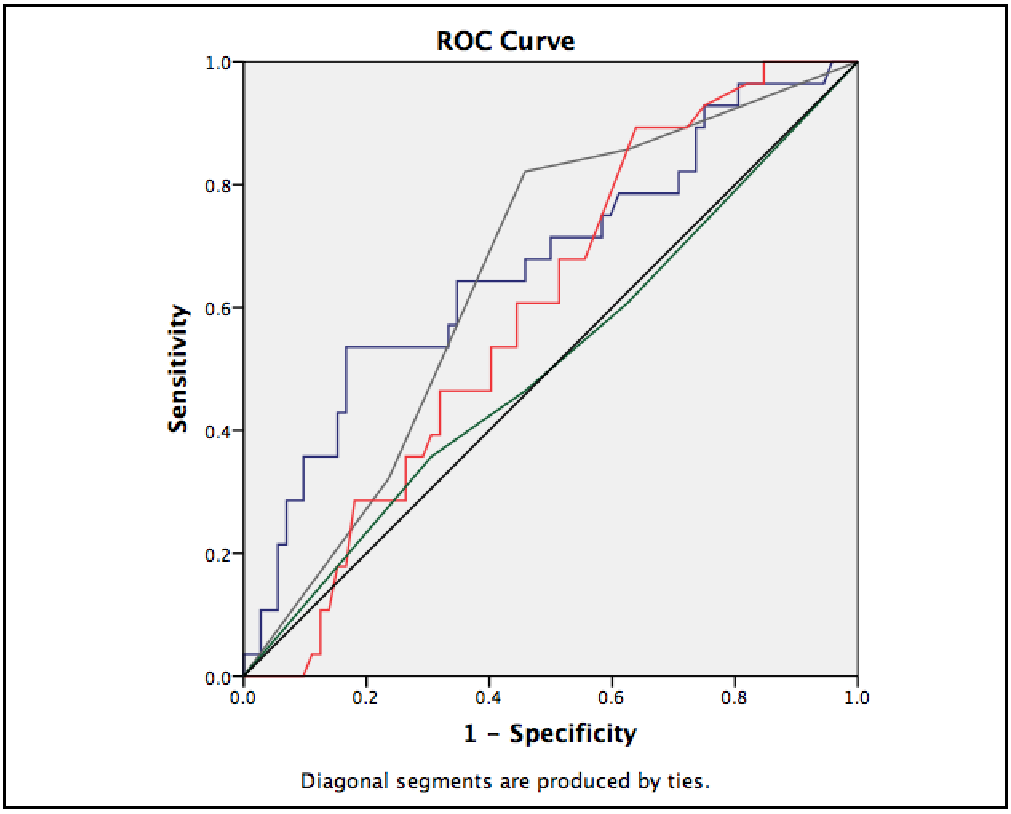

References: Red line = APACHE IV (AUC: 0.60, 95% CI: 0.48 – 0.71, *p* 0.136), green line = WFNS (AUC: 0.51, 95% CI: 0.38 – 0.64, *p* 0.902), grey line = Modified Fisher scale (AUC 0.65, 95% CI: 0.54 - 0.77, *p* 0.018), blue line = CT-proAVP (AUC: 0.67, 95% CI: 0.55 - 0.79, *p* 0.008) and black line = reference line.

Legends: APACHE IV: Acute physiology and chronic health evaluation IV, WFNS: World Federation of Neurological Surgeons score, CT-proAVP: C-terminal pro-arginine vasopressin.
